# Chest X‐ray severity score Brixia: From marker of early COVID‐19 infection to predictor of worse outcome in internal medicine wards

**DOI:** 10.1111/eci.13908

**Published:** 2022-11-21

**Authors:** Federico Carbone, Alessandro Casaleggio, Martina Fiannacca, Fabio Borda, Stefano Ministrini, Giulia Vischi, Valeria Carpaneto, Matteo Sobrero, Chiara Monti, Daria De Stefano, Benedetta Saccomanno, Marcella Massone, Arianna Piccardo, Alessandro Calvia, Federica Vischi, Maddalena Bagnasco, Ottavia Magnani, Matteo Caiti, Elisabetta Cenni, Paola Ballarino, Patrizia Giuntini, Alessandra Barreca, Chiara Tognoni, Federica Pirisi, Paolo Canepa, Domenico Cerminara, Lisa Pelanconi, Michele Strozzi, Amedeo Thneibat, Mario Stabile, Edineia Felix, Selena Dasso, Cecilia Casini, Alberto Minetti, Andrea Lorenzo Poggi, Roberta Gonella, Fabio Ferrando, Andrea Bellodi, Alberto Ballestrero, Paolo Barbera, Eleonora Arboscello, Aldo Pende, Paolo Moscatelli, Giuseppe Cittadini, Fabrizio Montecucco

**Affiliations:** ^1^ Department of Internal Medicine University of Genoa School of Medicine Genoa Italy; ^2^ IRCCS Ospedale Policlinico San Martino Genoa Genoa Italy; ^3^ Department of Health Sciences (DISSAL) – Radiology Section University of Genoa Genoa Italy; ^4^ Internal Medicine Department, "Santa Maria della Misericordia" Hospital University of Perugia Perugia Italy; ^5^ Center for Molecular Cardiology University of Zurich Schlieren Switzerland

**Keywords:** Brixia, chest X‐ray, COVID‐19, mortality, thrombosis

## Abstract

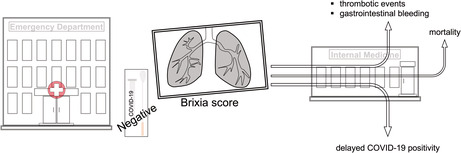

## 1 INTRODUCTION

Two years and half after the first COVID‐19 outbreak, we keep on learning about it. Never before in science history, so much information was quickly generated, shared and deployed. Such an amount of research and scientific development contributed to face off the challenges imposed by the pandemic that need now to be deeply dived into and not be forgotten. A growing literature is focusing on a post‐COVID‐19 pandemic—likely endemic—world, with a call for the next pandemic.^1^ Early in the forerunner countries, preventing and identifying early in‐hospital COVID‐19 positivity was one of the leading challenges and required a substantial step forward in hospital management and patient care.^2^, ^3^ Here, we focused on radiological assessment performed at ED admission during the first pandemic wave. Radiological imaging has indeed gained a critical role in the diagnosis of COVID‐19 patients. Especially chest X‐ray (CXR) has early become a useful diagnostic and monitoring tool, due to the feasibility in the emergency setting, despite its low specificity in the early stage of disease.^4^ Multiple CXR‐based scoring systems have been proposed to stratify the risk of disease progression and mortality.^5^, ^6^, ^7^ Here, we investigated whether the semi‐quantitative scoring of CXR Brixia may deserve a role beyond the early COVID‐19 positivity such as prediction of late COVID‐19 infection and serious adverse outcome (i.e. thrombotic complications and gastrointestinal [GI] bleeding).

## 2 METHODS

### 2.1 Patient enrolment and assessment

This is a sub‐analysis of a previously published retrospective study carried out at the IRCCS Ospedale Policlinico San Martino in Genoa during the first wave of COVID‐19 pandemic.^3^ Briefly, patients tested negative for COVID‐19 infection during stay at ED admission were enrolled from 24 February to 24 May 2020. Brixia score was then consecutively assessed in the first 283 enrolled patients (Figure S1). Clinical and biochemical data performed at ED admission have been collected from hospital records, as previously described.^3^ The present study was approved by the local ethics board of IRCCS Ospedale Policlinico San Martino (200/2020 – DB id 10,515). The study was carried out in accordance with The Code of Ethics of the World Medical Association (Declaration of Helsinki) for experiments involving humans and adhered to the principles of the STROBE statement.

### 2.2 Chest X‐ray scoring by Brixia

**FIGURE 1:**
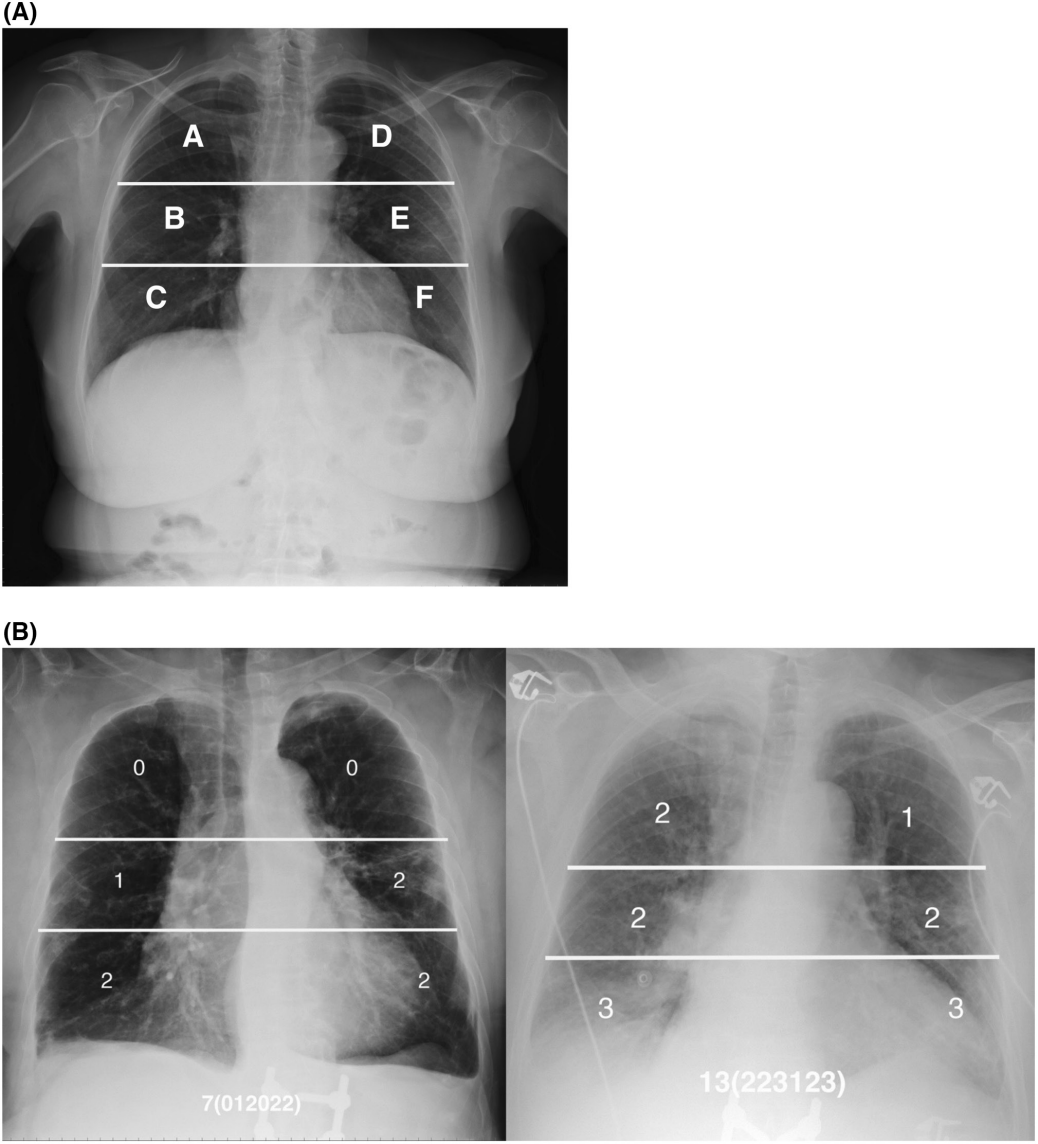
Brixia score method. The image summarizes lung division into three zones of Brixia score (A): upper (A and D), middle (B and E) and lower (C and F). Panel B summarizes Brixia lung scores across lung zones.

Brixia scoring system was calculated according with previous literature^4^, ^6^ (Figure 1). Chest radiographs were performed with DRX Revolution and Kodak DirectView DR9000 (both from Carestream Health) or SEDECAL—Radiologico Mobile Digitale (ATS), or Roller 15/30 (SMAM). Image interpretation was carried out by two expert radiologists, blinded to the other clinical variables and outcome, through SuitEstensa RIS (Esaote).

### 2.3 Study endpoints adjudication and sample size calculation

A case of delayed COVID‐19 positivity is defined by a patient tested positive for COVID‐19 infection in Internal Medicine wards after being negative during stay at ED. The primary outcome of the study is then to establish the predictive role of Brixia score towards delayed COVID‐19 positivity during hospital stay. As model with binary outcome, our sample size (>125) subjects satisfy the requirements for having a power greater than 80% and a type I error lower than 5% (see AppendixS1). As secondary outcomes, we consider any COVID‐19 positivity during stay in both ED and Internal Medicine ward, occurrence of adverse events (a composite of thrombotic complications and gastrointestinal [GI] bleeding) and overall mortality. The latter was tested after Brixia score categorization (< vs. ≥ 8), as previously reported.^4^

### 2.4 Statistical analysis

Analyses are performed with GraphPad Prism version 9.0.0 for Windows (GraphPad Software, San Diego, CA) and R environment for statistical computing (URL http://www. R‐project. org/). Categorical data are presented as absolute and relative frequencies, whereas continuous ones as median and interquartile range [IQR] since the normality assumption is not demonstrated. Unpaired intergroup comparisons are drawn by Fisher's exact test and Mann–Whitney *U‐*test, as appropriate. Spearman's rank coefficients are calculated to investigate the correlations between continuous/ordinal variables, whereas Cox proportional hazards models are built to test the predictive ability of Brixia scoring system towards any time and late (in‐ward) COVID‐19 positivity during hospitalization, and overall mortality as well. They are expressed as hazard ratio (HR) with 95% confidence interval (CI). For the latter, Brixia categorization also allows survival rate estimation through Log‐rank test and Kaplan–Meier curve. Logistic regression models (presented as odds ratio [OR] with 95% CI) are further built to address the predictive role of Brixia score towards adverse thrombotic events and GI bleeding. For the adjusted models, forward stepwise regression analysis is used and non‐normally variables are log‐transformed to meet the linearity requirement for regression. Performances of regression analyses are also tested for (i) calibration by Hosmer–Lemeshow goodness of fit test, (ii) discrimination through receiver operator characteristic (ROC) curve and (iii) internal validation by bootstrap resampling. For all statistical analyses, a 2‐sided *p*‐Value <0.05 was considered as statistically significant.

## 3 RESULTS

### 3.1 Delayed COVID‐19 positivity is associated with patients age and frailty and length of hospitalization

Clinical characteristics of the study cohort are summarized in Tables 1 and Tables S1,S2. As in the original cohort, patients are elderly with a median age of 80 years and equally distributed across sexes (52.1% of males). Patients with in‐hospital COVID‐19 positivity have more frequently fever (*p* = 0.03 and *p* = 0.099, respectively) and dyspnoea (*p* = 0.003 and *p* = 0.042, respectively) at ED admission. Since tested positive patients were immediately transferred to COVID‐19 dedicated units, their median length of stay in Internal Medicine ward was lower (5 days vs. 10 days, *p* = 0.007), without significant differences in the overall hospitalization time. Rather, the time to delayed in‐hospital positivity is correlated with advanced age (*p* = 0.006), comorbidity burden (*p* < 0.001), impaired renal function (*p* = 0.009 and 0.001 for creatinine and estimated glomerular filtration rate [eGFR], respectively) and inflammatory status (*p* = 0.037 for C‐reactive protein [CRP]) (Table S3).

**TABLE 1:** Clinical and anthropometric parameters of patients admitted at Emergency Department.

	Overall (*n* = 283)	Persistent negative (*n* = 265)	Delayed positivity (*n* = 18)	*p*‐Value
Age, years [IQR]	80 [72–87]	80 [72–87]	78 [71–86]	0.558
Sex, male (%)	149 (52.1)	129 (48.1)	10 (55.6)	0.813
Post‐menopausal women, yes (% among women)	144 (96.6)	134 (50.0)	10 (55.6)	0.543
Ethnicity				
Caucasian, *n* (%)	284 (99.3)	266 (99.3)	18 (100.0)	0.935
North Africans, *n* (%)	1 (0.3)	1 (0.4)	0 (0.0)	
Provenance				
Home, *n* (%)	262 (91.6)	246 (91.8)	16 (88.9)	0.678
Senior residence, *n* (%)	17 (5.9)	16 (6.0)	1 (5.6)	
Other, *n* (%)	7 (2.4)	6 (2.2)	1 (5.6)	
Contact with case				
None, *n* (%)	269 (94.1)	253 (94.4)	16 (88.9)	0.284
Suspected, *n* (%)	13 (4.5)	11 (4.1)	2 (11.1)	
Yes, *n* (%)	4 (1.4)	4 (1.5)	0 (0.0)	
Fever, yes (%)	90 (31.5)	**80 (29.9)**	**10 (55.6)**	**0.034**
Dyspnoea, yes (%)	53 (18.5)	**45 (16.8)**	**8 (44.4)**	**0.003**
Anosmia, yes (%)	101 (35.3)	98 (36.6)	3 (16.7)	0.093
Diarrhoea, yes (%)	3 (1.0)	3 (1.1)	0 (0.0)	0.652
Smokers				
Never, *n* (%)	173 (60.5)	160 (59.7)	13 (72.2)	0.301
Previous, *n* (%)	80 (28.0)	75 (28.0)	5 (27.8)	
Yes, *n* (%)	30 (10.5)	30 (11.2)	0 (0.0)	
Hypertension, yes (%)	187 (65.4)	177 (66.0)	10 (55.6)	0.440
Charlson comorbidity index [IQR]	6 [5–9]	6 [5–9]	7 [45–10]	0.992
Body temperature, C° [IQR]	36.5 [36.1–37.3]	36.5 [36.1–37.2]	36.8 [36.2–37.8]	0.320
Respiratory rate, acts/min [IQR]	18 [16–24]	18 [16–24]	20 [17–24]	0.672
Heart rate, bpm [IQR]	86 [75–100]	86 [75–99]	98 [76–129]	0.110
sBP, mmHg [IQR]	130 [115–150]	130 [116–150]	135 [100–153]	0.705
dBP, mmHg [IQR]	75 [65–84]	75 [65–85]	73 [68–81]	0.724
ED hospitalization length, days [IQR]	2 [1–4]	1[1–4]	3 [2–7]	0.077
In‐ward hospitalization length, days [IQR]	10 [6–15]	**10 [6–16]**	**5 [3–10]**	**0.007**
Overall hospitalization length, days [IQR]	13 [9–20]	13 [9–21]	11 [7–14]	0.119
Brixia score	2 [0–6]	**2 [0–5]**	**4 [0–10]**	**0.038**

*Note*: Continuous data are presented as median [interquartile range, IQR] whereas categorical ones as absolute (relative) count. The p‐Values were calculated with Mann–Whitney test or Fisher exact test, as appropriate.

Abbreviations: dBP, diastolic blood pressure; ED, emergency department; sBP, systolic blood pressure.

Significant values are in bold.

### 3.2 Brixia score is associated with clinical suspicion of COVID‐19 infection and in‐hospital positivity

**FIGURE 2:**
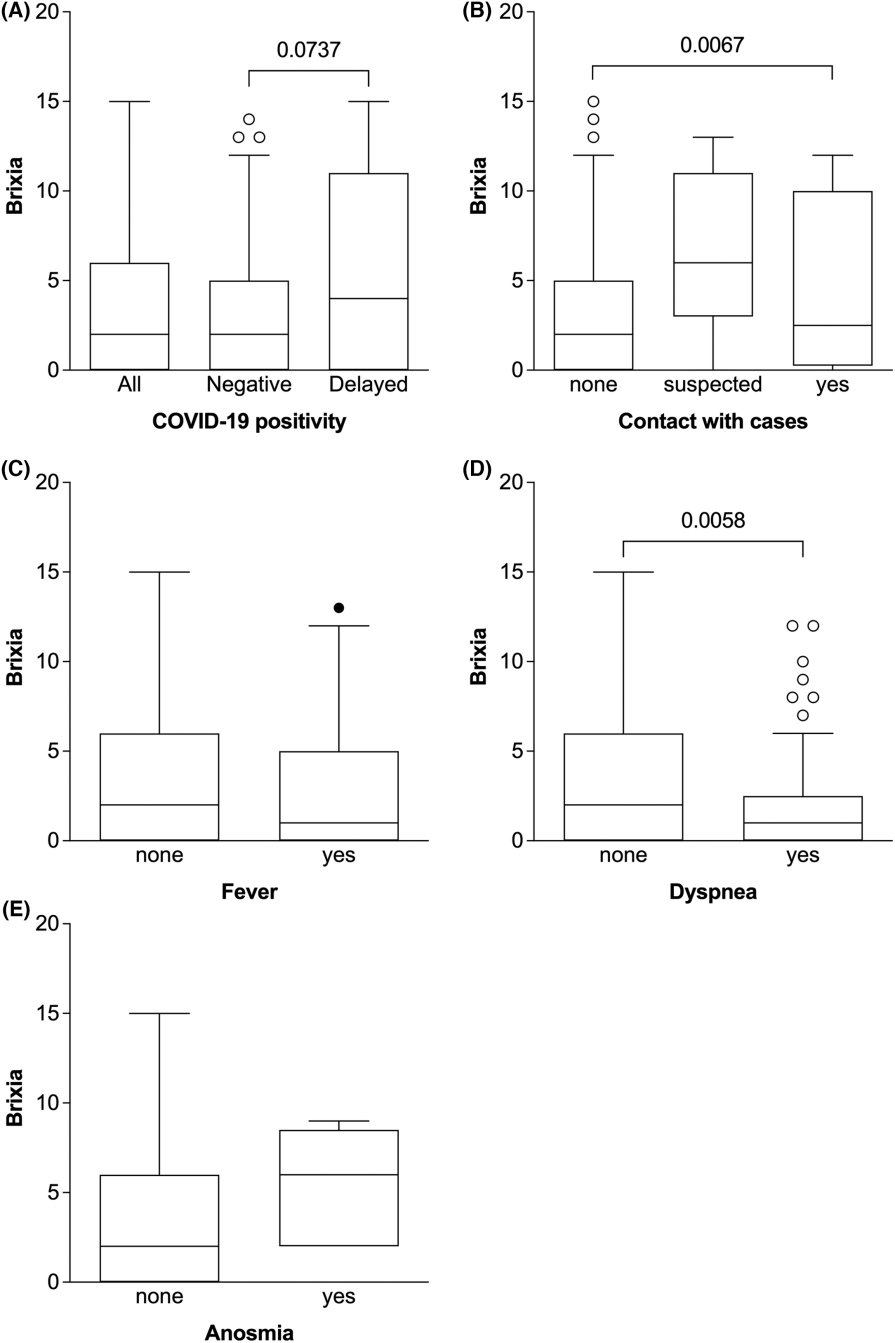
Box plot summarizing the main associations of Brixia with clinical variables. Values of Brixia are higher in patients with delayed at any time and late COVID‐19 positivity (A). Brixia values are also associated with the history of contact with suspected/certain cases of COVID‐19 infection (B), fever (C), dyspnoea (D) and anosmia (E).

At ED admission, Brixia score is higher in patients with delayed in‐hospital positivity (*p* = 0.0737), history of contact with COVID‐19 cases before hospitalization (*p*‐Value for trend 0.0067) and dyspnoea (*p* = 0.0058) (Figure 2A–E; Tables S4,S5).

### 3.3 Brixia score independently predicts delayed in‐hospital COVID‐19 positivity

**FIGURE 3:**
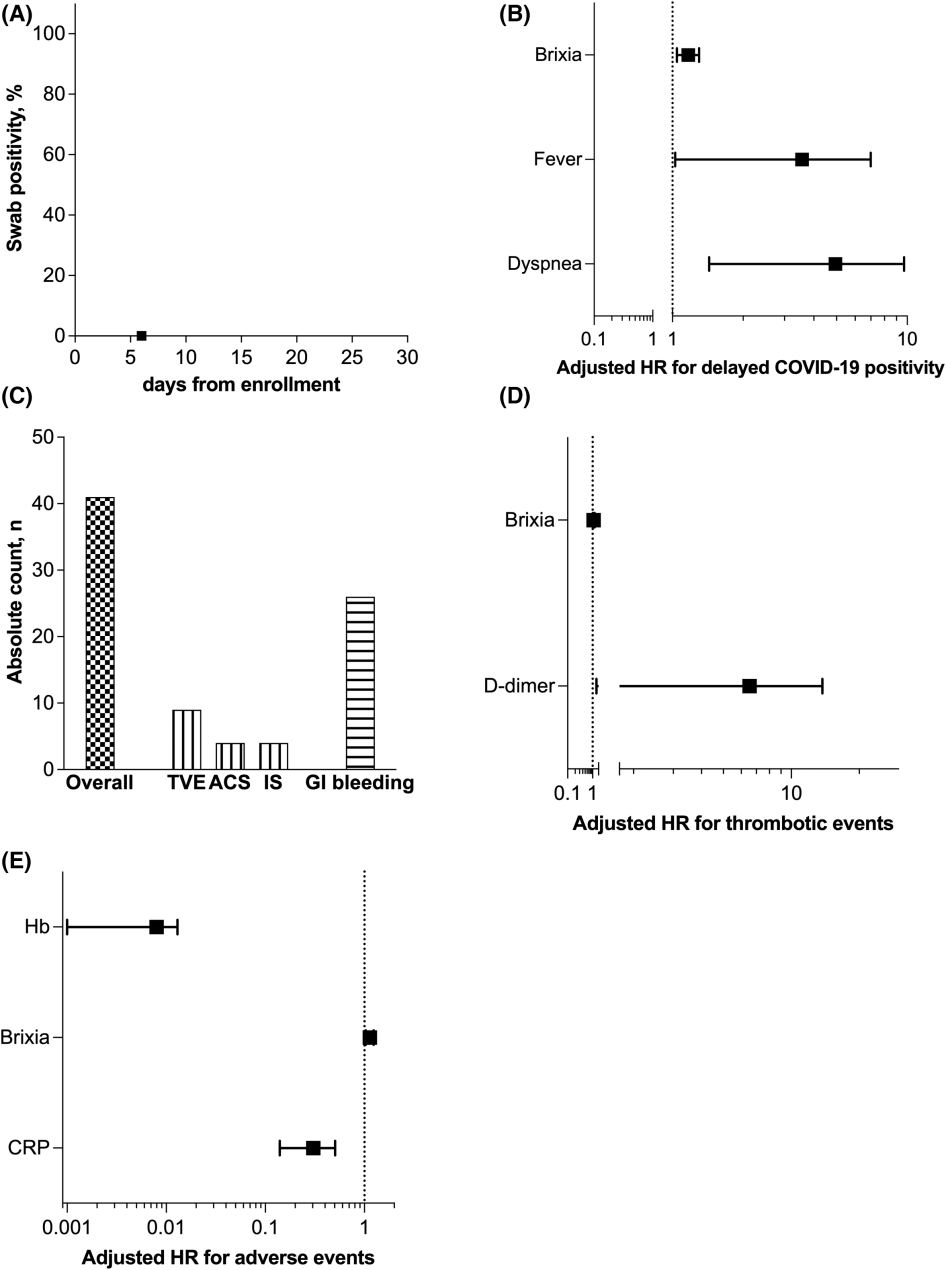
Brixia score predict adverse outcomes in patients hospitalized in Internal Medicine wards during the first wave of COVID‐19 pandemic in a forerunner country. Cumulative COVID‐19 positivity occurs early after hospital enrolment or admission in Internal medicine wards (A), and Brixia independently predicts this outcome in Cox proportional hazard regression models (B and C). The risk of thrombotic complications (i.e., venous thromboembolism [VTE], acute coronary syndrome [ACS], and and ischaemic stroke [IS]) and gastrointestinal (GI) bleeding (D) is independently associated with Brixia sore in logistic regression models.

Delayed in‐hospital positivity occurred in 18 patients (6.4%) of total cohort and within 5 days in the half of cases (Figure 3A). Of them, 7 (38.9%) occurred at the first test just upon the admission in COVID‐19‐free Internal Medicine Units (Figure 1). Brixia score shows a significant predictive value towards delayed in‐hospital COVID‐19 positivity (HR 1.124 [1.007–1.254]) (Table S6). It also fits into a model with fever and dyspnoea (HR 1.164 [1.044–1.299]) (Figure 3B; Table S6). With a *p*‐Value of 0.123, the Hosmer–Lemeshow test confirmed the good calibration of this model, whereas the result of ROC curve analysis indicated a significant discrimination performance of the model with an area under the curve of 0.765. As internal validation, the HR estimated from the original dataset fall within the new confidence intervals calculated with bootstrap resampling.

### 3.4 Brixia score independently predicts adverse outcomes in the whole study cohort

During hospitalization, forty patients suffer gastrointestinal bleeding (*n* = 26) and/or thrombotic complications (*n* = 17), namely venous/pulmonary thromboembolism (*n* = 9), acute coronary syndrome (*n* = 4) and ischaemic stroke (*n* = 4) (Figure 3C). Brixia scoring system shows an independent association with serious hospital complications, fitting a model with haemoglobin and CRP towards overall adverse event (OR 1.131 [1.032–1.239]) and with D‐dimer for thrombotic events (OR 1.344 [1.116–1.617]) (Figure 3D,E).

**FIGURE 4:**
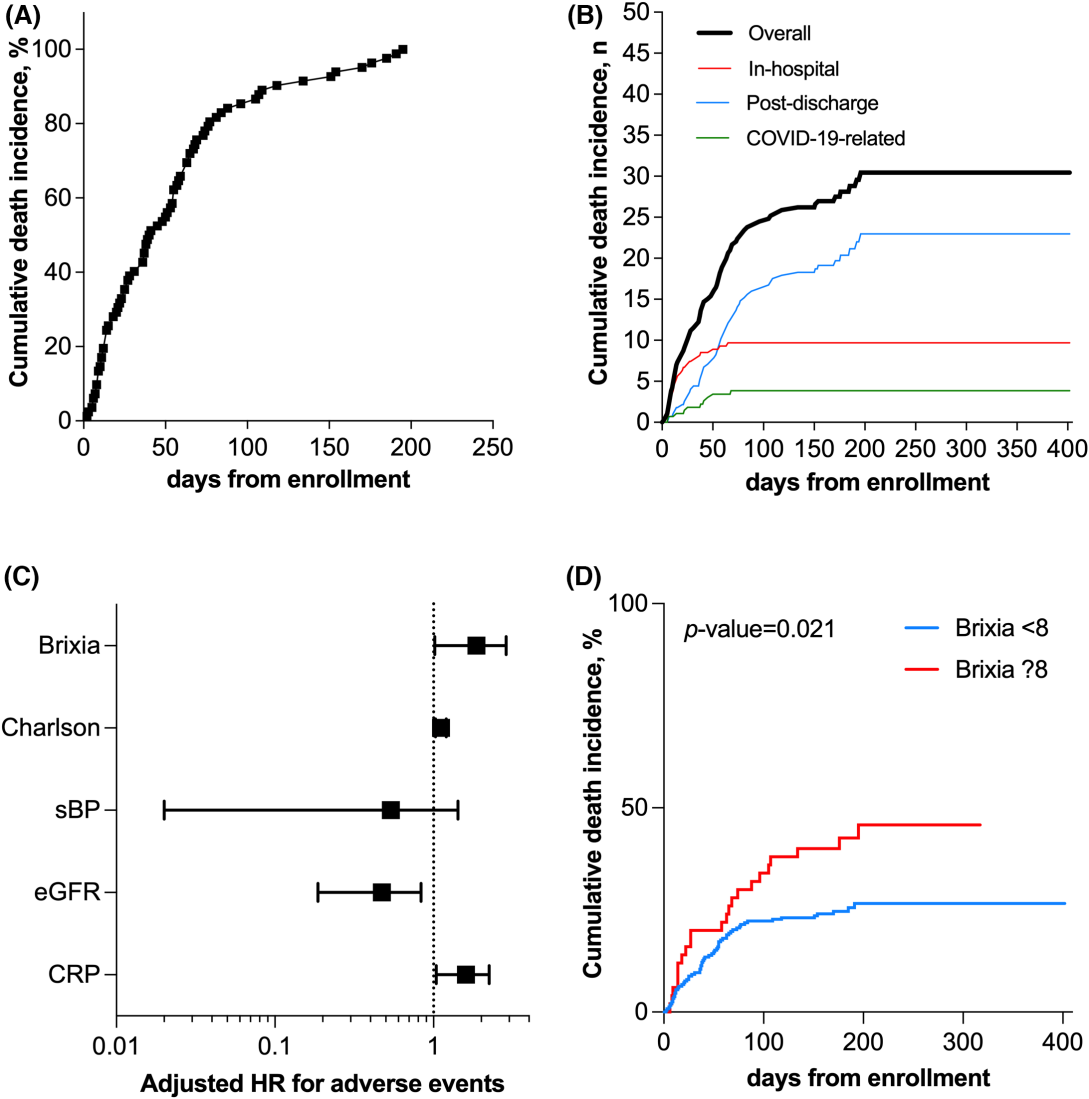
Brixia score predicts overall mortality in patients hospitalized in Internal Medicine wards during the first wave of COVID‐19 pandemic in a forerunner country. Cumulative mortality incidence and categorization across in‐hospital, post‐discharge and COVID‐19‐realted are summarized in panel A and B. Once categorized for values ≥8, Brixia independently predicts mortality in a Cox proportional hazard regression model (C). Kaplan–Meier curve with Log Rank test confirmed this associations (D).

Death was recorded in 81 patients (28.6%) during a median follow‐up of 169 days (range 2 to 402 days). In‐hospital death occurred in 27 patients with only 10 cases directly related to the COVID‐19 infection (Figure 4A,B). Once categorized, Brixia values ≥8 are independent predictors of overall mortality (HR 1.948 [1.194–3.176]) and fit a model with Charlson comorbidity index, systolic blood pressure, eGFR and CRP (HR 1.708 [1.018–2.868]) (Figure 4C). Kaplan–Meier survival curve confirmed the higher mortality risk associated with a Brixia score ≥8 with a *p*‐Value at log rank test of 0.021 (Figure 4D).

## 4 DISCUSSION

During the first wave of pandemic, many diagnostic pitfalls challenged clinicians in their threshold of suspicion for COVID‐19 infection. Early, reliable and feasible tools for detecting COVID‐19 early at ED admission were missing and not easy to implement.^8^ Here, we point out the role of the semi‐quantitative CXR score Brixia on COVID‐19 diagnosis/prognosis^4^, ^6^ and beyond. The association with history of contact with suspicious/certain cases and the hallmark symptoms of COVID‐19 infection might support the supremacy of clinical/radiological diagnosis towards molecular positivity. Nasopharyngeal swabs were routinely repeated during ED stay and this make consistent the hypothesis of a greater predictive power of Brixia, at least compared with the first‐generation molecular tests for COVID‐19. As additional finding we here report a predictive value of Brixia towards death and atherothrombotic adverse events during hospitalization and post‐discharge. The fitting with d‐dimer in logistic regression model would also suggest a pathophysiological explanation. Similar to previous coronavirus pandemics (i.e. SARS‐CoV‐1 and MERS‐CoV‐1), the association of SARS‐CoV‐2 infection with D‐dimer and coagulation abnormalities is clearly established.^9^ Endothelial dysfunction and microvascular thrombosis are well‐described mechanisms by which COVID‐19 infection would trigger venous thromboembolism/pulmonary embolism and complement‐mediated thrombotic microangiopathies/disseminated intravascular coagulation.^10^ Whether thrombotic complications also underlie the previous reported association between Brixia score and clinical worsening (i.e. non‐invasive ventilation, intubation and/or admission in intensive care unit) cannot be ruled out.^7^

Even more intriguingly, we observed this association in the whole cohort that include a prevalence of COVID‐19 negative patients. This should pave the way for future studies addressing clinical features associated with these radiological patterns. The independent association with mortality is another intriguing finding. Here, we confirm a greater mortality risk in elderly and frail patients with high comorbidity burden—mainly cardiovascular and renal—and inflammatory status^6^, ^11^ Whether Brixia score is ‘the pitch of the iceberg’ that reflect underlying pathological conditions remains to be elucidated.

Overall, semi‐quantitative scoring of CXR by Brixia would display a relevant prognostic role, not limited to COVID‐19 infection. It would also have a supremacy over CT scan, a measure burdened by high radiation dose, longer examination time and less feasibility in ED^12^ and peripheral healthcare centres.^13^ The lessons from the COVID‐19 pandemic may then prepare for further waves, new pandemics and open new application for the Brixia scoring systemic as well. CXR indeed meets the need to merge radiological, demographic, biochemical and clinical features in the shortest time, optimizing patient diagnosis/risk stratification and reducing the person‐to‐person transmission.^14^ Information from Brixia score might also be extended through radiomic analysis and/or artificial intelligence.^15^

As sub‐analysis of a larger retrospective cohort, this study has an intrinsic limitation, although the sample analysed is representative of the original cohort and they both share the primary outcome. Conversely, this—and the whole cohort—may be not representative of the global pandemic, being the recruitment time limited to the first wave of pandemic in a forerunner county (i.e. Italy). As above‐mentioned, the better performance of Brixia score as compared to a swab test for COVID‐19 need validation with later molecular/antigenic kits. Similarly, external validations with other pandemic waves where virus variants overlapped, and vaccinated people increased would be appropriate. In conclusion, this sub‐analysis of retrospective cohort points out the role of chest X‐ray scoring with Brixia as predictive tool of delayed COVID‐19 positivity. This would have a relevant impact in preserving COVID‐19‐free wards from in‐hospital cluster of contagion. Results from secondary outcomes (i.e. thrombotic complication and long‐term overall mortality) also suggest potential future applications of Brixia score beyond COVID‐19 pandemic. This would deserve specifically designed studies so that new tool may develop from the pandemic wave.

## CONFLICT OF INTEREST

The authors report no relationships that could be construed as a conflict of interest.

## Supporting information


Appendix S1:

## Data Availability

The datasets used and/or analysedanalyzed during the current study are available from the corresponding author on reasonable request.
