# Supplementary material for: Chest X‐ray severity score Brixia: From marker of early COVID‐19 infection to predictor of worse outcome in internal medicine wards
Source: Eur J Clin Invest. 2022 Nov 21;53(2):e13908. doi: 10.1111/eci.13908 (PMC10078553; doi:10.1111/eci.13908)
Supplement: Supplementary file 1 — Appendix S1: [file ECI-53-0-s001.docx]

# Supplementary Material

**Figure S1. Flow diagram illustrating patient selection**

# Table S1. Comorbidities (Charlson comorbidity index) at admission.

|  | **Overall**  **(n=286)** | **Persistent negative**  **(n=268)** | **Delayed positivity**  **(n=18)** | ***p*-value** |
| --- | --- | --- | --- | --- |
| History of myocardial infarction, yes (%) | 62 (21.7) | 58 (21.9) | 4 (22.2) | 0.973 |
| Chronic heart failure, yes (%) | 107 (37.4) | 100 (37.3) | 7 (38.9) | 0.932 |
| Peripheral vascular disease, yes (%) | 51 (17.8) | 48 (18.4) | 3 (16.7) | 0.824 |
| History of TIA/stroke, yes (%) | 40 (14.0) | 36 (13.4) | 14 (22.2) | 0.312 |
| Dementia, yes (%) | 61 (21.3) | 58 (21.9) | 3 (16.7) | 0.578 |
| COPD, yes (%) | 64 (22.4) | 61 (22.8) | 3 (16.7) | 0.533 |
| Connective tissue disease, yes (%) | 7 (2.4) | 7 (2.6) | 0 (0.0) | 0.485 |
| History of peptic ulcer disease, yes (%) | 4 (1.4) | **2 (0.8)** | **2 (11.1)** | **<0.001** |
| Liver disease |  |  |  | 0.822 |
| None, n (%) | 269 (94.1) | 252 (95.1) | 17 (94.4) |  |
| Mild, n (%) | 8 (2.8) | 7 (2.6) | 1 (5.6) |  |
| Moderate, n (%) | 2 (0.7) | 2 (0.8) | 0 (0.0) |  |
| Severe, n (%) | 4 (1.4) | 4 (1.5) | 0 (0.0) |  |
| Diabetes |  |  |  | 0.374 |
| None or under diet control, n (%) | 209 (73.1) | 198 (73.9) | 11 (61.1) |  |
| Uncomplicated (%) | 47 (16.4) | 42 (15.7) | 5 (27.8) |  |
| End-organ damage, n (% | 25 (8.7) | 23 (8.6) | 2 (11.1) |  |
| Hemiplegia, yes (%) | 5 (1.7) | 4 (1.5) | 1 (5.6) | 0.329 |
| Moderate-to-severe CKD, yes (%) | 56 (19.6) | 55 (20.8) | 1 (5.6) | 0.228 |
| Solid malignancy, yes (%) | 54 (18.9) | 50 (18.8) | 4 (22.2) | 0.720 |
| Leukemia, yes (%) | 6 (2.1) | 5 (1.9) | 1 (5.6) | 0.294 |
| Lymphoma, yes (%) | 5 (1.7) | 5 (1.9) | 0 (0.0) | 0.557 |
| AIDS, yes (%) | 0 (0.0) | 0 (0.0) | 0 (0.0) | – |

Data are presented as absolute (relative) count. The p-values were calculated with Mann-Whitney test or Fisher exact test, as appropriate.

TIA: transient ischemic attack; COPD: chronic obstructive pulmonary disease; CKD: chronic kidney disease; AIDS: acquired immunodeficiency syndrome.

# Table S2. Laboratory findings characterizing patients admitted at Emergency Department.

|  | **Overall**  **(n=286)** | **Persistent negative**  **(n=268)** | **Delayed positivity**  **(n=18)** | ***p*-value** |
| --- | --- | --- | --- | --- |
| SaO2, % [IQR] | 95 [91 – 97] | 95 [91 – 97] | 93 [90 – 96] | 0.490 |
| pO2, mmHg [IQR] | 70 [59 – 87] | 70 [60 – 87] | 73 [57 – 87] | 0.741 |
| P/F [IQR] | 298 [243 – 364] | 298 [244 – 364] | 293 [219 – 363] | 0.707 |
| pCO2, mmHg [IQR] | 36 [33 – 40] | 35 [33 – 39] | 37 [34 – 41] | 0.843 |
| HCO3^-^ mmol/mol [IQR] | 24 [21 – 27] | 24 [21 – 26] | 26 [22 – 29] | 0.346 |
| Lactates mmol/L [IQR] | 1.2 [0.9 – 1.9] | **1.2 [0.9 – 1.9]** | 1.1 [0.5 – 1.8] | 0.414 |
| Hb, g/dL [IQR] | 12.4 [10.3 – 13.8] | 12.4 [10.4 – 13.9] | 12.0 [9.5 – 13.2] | 0.360 |
| WBC, cells x 10^3^/mm3 [IQR] | 9.9 [7.0 – 13.2] | 9.8 [7.1 – 13.1] | 11.5 [5.9 – 17.7] | 0.538 |
| Neutrophil count, cells/mm3 [IQR] | 7.8 [5.2 – 10.8] | 7.7 [5.3 – 10.7] | 8.9 [4.2 – 14.6] | 0.608 |
| Platelets, cells x 10^3^/mm3 [IQR] | 243 [171 – 322] | 242 [170 – 322] | 278 [183 – 323] | 0.573 |
| INR [IQR] | 1.25 [1.12 – 1.50] | 1.25 [1.12 – 1.60] | 1.31 [1.11 – 1.46] | 0.917 |
| aPTT, sec [IQR] | 32 [29 – 36] | 32 [29 – 36] | 32 [31 – 34] | 0.643 |
| Fibrinogen, g/L [IQR] | 4.1 [2.6 – 5.8] | 3.7 [2.2 – 5.6] | 7.6 [5.0 – 8.1] | 0.056 |
| D-dimer | 1626 [926 – 4821] | 1643 [935 – 5092] | 1094 [795 – 2470] | 0.144 |
| Creatinine, mg/dL [IQR] | 1.1 [0.8 – 1.7] | 1.1 [0.8 – 1.7] | 0.9 [0.6 – 1.7] | 0.302 |
| eGFR, mL/min [IQR] | 56 [34 – 79] | 56 [33 – 78] | 56 [35 – 88] | 0.482 |
| Total bilirubin, mg/dL [IQR] | 0.60 [0.41 – 0.90] | 0.61 [0.42 – 0.91] | 0.49 [0.37 – 0.90] | 0.169 |
| CRP, mg/L [IQR] | 30 [6 – 90] | 28 [6 – 86] | 50 [21 – 182] | 0.076 |

Data are presented as median [interquartile range]. The p-values were calculated with Mann-Whitney test or Fisher exact test, as appropriate.

SaO2. O2 saturation, pO2: pressures of oxygen; P/F: pressure of oxygen/fractional inspired oxygen ratio; pO2: pressures of carbon dioxide; HCO3^-^: bicarbonate; Hb: hemoglobin, WBC: white blood cells; PT: prothrombin time; INR: international normalized ratio; aPTT: activated partial thromboplastin time; eGFR: estimated glomerular filtration rate; LDH: Lactate dehydrogenase; CRP: C-reactive protein.

# Table S3. Correlation of clinical and biochemical variables with the time to positivity.

|  | **Time to positivity** | |
| --- | --- | --- |
|  | ***ρ*** | ***p*-value** |
| Age | **0.166** | **0.005** |
| Charlson comorbidity index | **0.210** | **<0.001** |
| Weight | 0.086 | 0.366 |
| Body temperature | -0.001 | 0.991 |
| Respiratory rate | 0.021 | 0.799 |
| Heart rate | -0.036 | 0.554 |
| sBP | -0.075 | 0.211 |
| dBP | -0.084 | 0.161 |
| ED hospitalization length | **0.368** | **<0.001** |
| In-ward hospitalization length | **0.729** | **<0.001** |
| Overall hospitalization length | **0.957** | **<0.001** |
| SaO2 | 0.045 | 0.611 |
| pO2 | 0.062 | 0.469 |
| P/F | -0.081 | 0.346 |
| pCO2 | 0.009 | 0.917 |
| HCO3 | 0.021 | 0.837 |
| Lactates | -0.048 | 0.640 |
| Hb | **-0.150** | **0.012** |
| WBC | 0.029 | 0.625 |
| Neutrophil count | 0.050 | 0.412 |
| Platelets | 0.091 | 0.129 |
| INR | -0.084 | 0.170 |
| aPTT | 0.073 | 0.228 |
| Fibrinogen | 0.142 | 0.416 |
| Creatinine | **0.157** | **0.009** |
| eGFR | **-0.192** | **0.001** |
| Total bilirubin | 0.019 | 0.757 |
| CRP | **0.127** | **0.037** |

The *p*-values refer to Spearman Rank correlation tests.

sBP: systolic blood pressure; dBP: diastolic blood pressure; ED: emergency department; SaO2. O2 saturation, pO2: pressures of oxygen; P/F: pressure of oxygen/fractional inspired oxygen ratio; pO2: pressures of carbon dioxide; HCO3^-^: bicarbonate; Hb: hemoglobin, WBC: white blood cells; PT: prothrombin time; INR: international normalized ratio; aPTT: activated partial thromboplastin time; eGFR: estimated glomerular filtration rate; CRP: C-reactive protein.

# Table S4. Variations in time to positivity and Brixia score according with clinical presentation.

|  | **Brixia score** | ***p*-value** |
| --- | --- | --- |
| Sex |  |  |
| Man | 2 [0 – 4] | 0.153 |
| Woman | 2 [0 – 6] |  |
| Hormonal status (among women), |  | **0.032** |
| Pre-menopausal status | 0 [0 – 1] |  |
| Post-menopausal women | 2 [0 – 6] |  |
| Ethnicity |  | 0.530 |
| Caucasian | 2 [0 – 6] |  |
| North Africans | 2 [2 – 2] |  |
| Provenance |  | 0.083 |
| Home | 2 [2 – 5] |  |
| Senior residence | 5 [1 – 10] |  |
| Other | 0 [0 – 3] |  |
| Contact with case |  | **0.003** |
| None | 2 [0 – 5] |  |
| Suspected | 6 [3 – 11] |  |
| Yes | 3 [0 – 10] |  |
| Fever, yes (%) |  | 0.099 |
| None | 2 [0 – 6] |  |
| Yes | 1 [0 – 5] |  |
| Dyspnea, yes (%) |  | **0.015** |
| None | **2 [0 – 6]** |  |
| Yes | **1 [0 – 3]** |  |
| Anosmia, yes (%) |  | **0.011** |
| None | **1 [0 – 4]** |  |
| Yes | **2 [0 – 6]** |  |
| Diarrhea, yes (%) |  |  |
| None | 1 [0 – 4] | 0.138 |
| Yes | 2 [0 – 6] |  |
| Smokers |  |  |
| None | 2 [0 – 5] | 0.620 |
| Previous | 3 [0 – 6] |  |
| Yes | 2 [0 – 7] |  |
| Hypertension, yes (%) |  |  |
| None | 2 [0 – 4] | 0.157 |
| Yes | 2 [0 – 6] |  |

Continuous data are presented as median [interquartile range] whereas categorical ones as absolute (relative) count. The *p*-values refers to the comparison between persistent negative and any positivity occurring during hospitalization (Mann-Whitney test or Kruskal-Wallis tests, as appropriate).

sBP: systolic blood pressure; dBP: diastolic blood pressure; ED: emergency department.

# Table S5. Correlations of Brixia score with clinical and biochemical variables.

|  | **Brixia score** | |
| --- | --- | --- |
|  | ***ρ*** | ***p*-value** |
| Age | 0.109 | 0.066 |
| Charlson comorbidity index | 0.099 | 0.095 |
| Weight | -0.021 | 0.822 |
| Body temperature | -0.117 | 0.053 |
| Respiratory rate | 0.006 | 0.940 |
| Heart rate | -0.078 | 0.202 |
| sBP | 0.046 | 0.446 |
| dBP | 0.039 | 0.517 |
| ED hospitalization length | 0.041 | 0.493 |
| In-ward hospitalization length | -0.034 | 0.568 |
| Overall hospitalization length | 0.022 | 0.706 |
| SaO2 | -0.126 | 0.150 |
| pO2 | -0.021 | 0.809 |
| P/F | -0.039 | 0.647 |
| pCO2 | -0.011 | 0.898 |
| HCO3 | 0.013 | 0.900 |
| Lactates | 0.063 | 0.537 |
| Hb | -0.010 | 0.865 |
| WBC | -0.005 | 0.938 |
| Neutrophil count | -0.001 | 0.982 |
| Platelets | -0.003 | 0.957 |
| INR | 0.012 | 0.848 |
| aPTT | 0.023 | 0.708 |
| Fibrinogen | 0.014 | 0.937 |
| Creatinine | 0.005 | 0.933 |
| eGFR | -0.079 | 0.191 |
| Total bilirubin | -0.039 | 0.530 |
| CRP | -0.014 | 0.813 |
| Time to positivity | -0.002 | 0.978 |

The *p*-values refer to Spearman Rank correlation tests.

sBP: systolic blood pressure; dBP: diastolic blood pressure; ED: emergency department; SaO2. O2 saturation, pO2: pressures of oxygen; P/F: pressure of oxygen/fractional inspired oxygen ratio; pO2: pressures of carbon dioxide; HCO3^-^: bicarbonate; Hb: hemoglobin, WBC: white blood cells; PT: prothrombin time; INR: international normalized ratio; aPTT: activated partial thromboplastin time; eGFR: estimated glomerular filtration rate; CRP: C-reactive protein.

# Table S6. Regression models assessing the predictive value/independent association of Brixia score.

|  | **Univariate** |  | **Adjusted** |  |
| --- | --- | --- | --- | --- |
| **Delayed swab positivity** | **HR (95% CI)** | ***p*-value** | **HR (95% CI)** | ***p*-value** |
| Brixia score | 1.124 (1.007 – 1.254) | **0.037** | 1.164 (1.044 – 1.299) | **0.006** |
| Age | 0.069 (0.001 – 4.012) | 0.197 |  |  |
| Fever (Ref=none) | 2.726 (1.075 – 6.911) | **0.035** | 2.680 (1.027 – 6.995) | **0.044** |
| Dyspnea (Ref=none) | 3.629 (1.431 – 9.199) | **0.007** | 3.726 (1.432 – 9.694) | **0.007** |
| Anosmia |  |  |  |  |
| Charlson comorbidity index | 0.977 (0.832 – 1.148) | 0.779 |  |  |
| Hb | 0.972 (0.819 – 1.154) | 0.744 |  |  |
| D-dimer | 0.348 (0.089 – 1.358) | 0.129 |  |  |
| eGFR | 2.591 (0.406 – 16.555) | 0.314 |  |  |
| CRP | 1.909 (0.864 – 4.220) | 0.110 |  |  |
|  |  |  |  |  |
| **Adverse events** | **OR (95% CI)** | ***p*-value** | **OR (95% CI)** | ***p*-value** |
| Brixia score | 1.009 (1.022 – 1.204) | **0.014** | 1.131 (1.032 – 1.239) | **0.008** |
| Dyspnea | 0.308 (0.091 – 1.038) | 0.058 |  |  |
| Anosmia |  |  |  |  |
| Hb | 0.010 (0.001 – 0.023) | **<0.001** | 0.01 (0.001 – 0.013) | **<0.001** |
| CRP | 0.326 (0.184 – 0.577) | **<0.001** | 0.266 (0.140 – 0.507) | **<0.001** |
|  |  |  |  |  |
| **Thrombotic events** | **OR (95% CI)** | ***p*-value** | **OR (95% CI)** | ***p*-value** |
| Brixia score | 1.200 (1.070 – 1.347) | **0.002** | 1.344 (1.116 – 1.617) | **0.008** |
| D-dimer | 4.444 (1.439 – 13.724) | **<0001** | 7.128 (1.824 – 27.861) | **0.005** |
|  |  |  |  |  |
| **GI bleeding** |  |  |  |  |
| Brixia score | 1.062 (0.959 – 1.175) | 0.247 |  |  |
|  |  |  |  |  |
| **Overall mortality** | **HR (95% CI)** | ***p*-value** | **HR (95% CI)** | ***p*-value** |
| Brixia score ≥8 | 1.948 (1.194 – 3.176) | **0.008** | 1.708 (1.018 – 2.868) | **0.043** |
| Age | 425.249 (7.651 – 2.364x10^4^) | **0.003** | 93.972 (0.848 – 1.042 x10^4^) | 0.059 |
| Charlson comorbidity index | 1.168 (1.089 – 1.253) | **<0.001** | 1.114 (1.029 – 1.205) | **0.008** |
| sBP | 0.005 (0.000 – 0.051) | **<0.001** | 0.004 (0.001 – 0.099) | **0.001** |
| dBP | 0.44 (0.005 – 0.424) | **0.007** | 7.273 (0.256 – 2.067x10^2^) | 0.245 |
| Hb | 0.088 (0.015 – 0.516) | **0.007** | 0.169 (0.020 – 1.424) | 0.102 |
| eGFR | 0.203 (0.112 – 0.366) | **<0.001** | 0.395 (0.187 – 0.834) | **0.015** |
| CRP | 1.705 (1.202 – 2.418)) | **0.003** | 1.528 (1.041 – 2.244) | **0.030** |

According with the guidelines for sample size calculation (<https://www.bmj.com/content/368/bmj.m441>), for a binary outcome:

- 1. In the case of models with binary outcome, a is recommended. This requires at least **125** subjects (Equation 1, Figure 1 of (Riley et al, 2020).

- 1. Models with binary outcome, a Mean Absolute Precision Error (MAPE) < 0.05 is recommended. Given an overall outcome proportion of 0.5, and having 3 predictors, at least **92** subjects are required in order to obtain the desired MAPE (Equation 2, Figure 2 of (Riley et al, 2020), with Φ=0.06, P=3, MAPE = 0.05).

- 1. In the case of models with binary outcome, an expected uniform shrinkage factor <10% is recommended. Having 3 candidate predictors, at least **254** subjects are required, with P=3, S=0.9, R^2^_cs_=0.1.

- 1. The model’s proportion of overall variance explained (R^2^_cs_) requires to be pre-specified in order to compute the expected uniform shrinkage factor (see point c). According to Figure 3 and 4 of (Riley et al, 2020), we select R^2^_cs_=0.1.


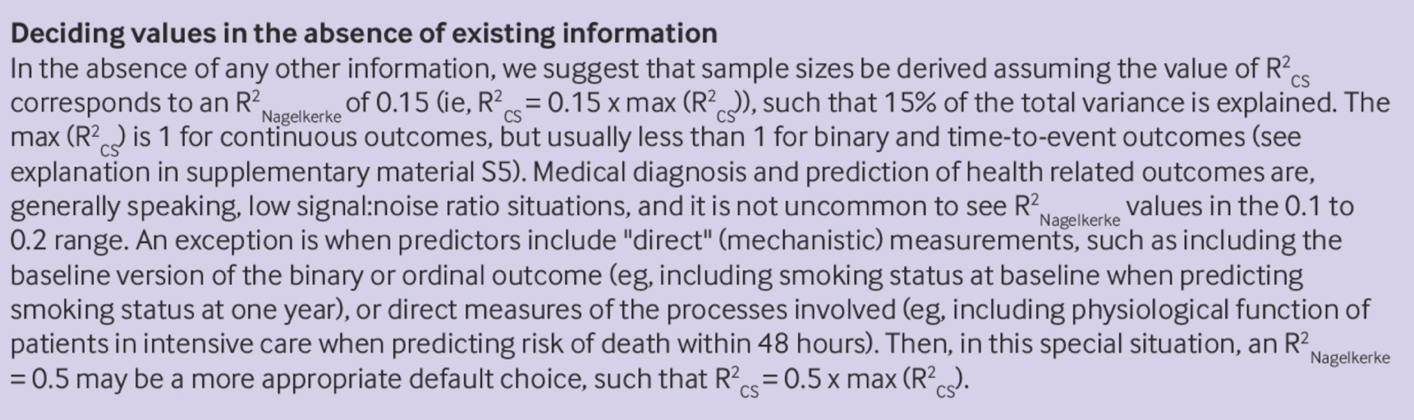


- 1. **Model calibration** performances are evaluated via the Hosmer-Lemeshow *(Hosmer, David W.; Lemeshow, Stanley (2013). Applied Logistic Regression. New York: Wiley.* [*ISBN*](https://en.wikipedia.org/wiki/ISBN_(identifier)) [*978-0-470-58247-3*](https://en.wikipedia.org/wiki/Special:BookSources/978-0-470-58247-3)*;* Bilder, Christopher R.; Loughin, Thomas M. (2014), Analysis of Categorical Data with R (First ed.), Chapman and Hall/CRC, [*ISBN*](https://en.wikipedia.org/wiki/ISBN_(identifier)) [*978-1439855676*](https://en.wikipedia.org/wiki/Special:BookSources/978-1439855676)*)* goodness of fit test with 10 groups. The returned p-value is p=0.123 with a χ square of 11.376, which indicates a good calibration of the model.
  2. **Model discrimination** performances are already presented via the ROC curve analysis (Table S4). The curve is presented below and shows that the model has high sensitivity and specificity simultaneously, therefore high discrimination ability.

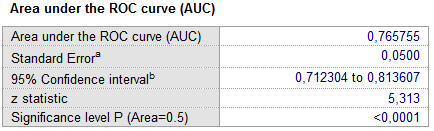


- 1. **Bootstrap resampling performance**.

Based on 1000 bootstrap replicates, we obtained the following estimate of the HR (average of the 1000 HRs from the 1000 bootstrap samples) and of their 95% confidence interval. When the HR estimated from the original dataset are considered, we note that they fall within the new bootstrap confidence intervals.

Brixia score HR 1.165 [1.019 – 1.344]

Fever HR 2.680 [1.002 – 8.178]

Dyspnea HR 3.719 [1.121 – 11.734]

**STROBE Statement—checklist of items that should be included in reports of observational studies**

|  | Item No | Recommendation | ✔(N/A) |
| --- | --- | --- | --- |
| **Title and abstract** |  |  |  |
|  | 1 | (*a*) Indicate the study’s design with a commonly used term in the title or the abstract | ✔ |
|  |  | (*b*) Provide in the abstract an informative and balanced summary of what was done and what was found | ✔ |
| Introduction | | |  |
| Background/rationale | 2 | Explain the scientific background and rationale for the investigation being reported | ✔ |
| Objectives | 3 | State specific objectives, including any prespecified hypotheses | ✔ |
| Methods | | |  |
| Study design | 4 | Present key elements of study design early in the paper | ✔ |
| Setting | 5 | Describe the setting, locations, and relevant dates, including periods of recruitment, exposure, follow-up, and data collection | ✔ |
| Participants | 6 | (*a*) *Cohort study*—Give the eligibility criteria, and the sources and methods of selection of participants. Describe methods of follow-up  *Case-control study*—Give the eligibility criteria, and the sources and methods of case ascertainment and control selection. Give the rationale for the choice of cases and controls  *Cross-sectional study*—Give the eligibility criteria, and the sources and methods of selection of participants | ✔ |
|  |  | (*b*) *Cohort study*—For matched studies, give matching criteria and number of exposed and unexposed  *Case-control study*—For matched studies, give matching criteria and the number of controls per case | N/A |
| Variables | 7 | Clearly define all outcomes, exposures, predictors, potential confounders, and effect modifiers. Give diagnostic criteria, if applicable | ✔ |
| Data sources/ measurement | 8* | For each variable of interest, give sources of data and details of methods of assessment (measurement). Describe comparability of assessment methods if there is more than one group | ✔ |
| Bias | 9 | Describe any efforts to address potential sources of bias | ✔ |
| Study size | 10 | Explain how the study size was arrived at | ✔ |
| Quantitative variables | 11 | Explain how quantitative variables were handled in the analyses. If applicable, describe which groupings were chosen and why | ✔ |
| Statistical methods | 12 | (*a*) Describe all statistical methods, including those used to control for confounding | ✔ |
|  |  | (*b*) Describe any methods used to examine subgroups and interactions | ✔ |
|  |  | (*c*) Explain how missing data were addressed | N/A |
|  |  | (*d*) *Cohort study*—If applicable, explain how loss to follow-up was addressed  *Case-control study*—If applicable, explain how matching of cases and controls was addressed  *Cross-sectional study*—If applicable, describe analytical methods taking account of sampling strategy | ✔ |
|  |  | (*e*) Describe any sensitivity analyses | ✔ |

| Results | | | ✔ (N/A) |
| --- | --- | --- | --- |
| Participants | 13* | (a) Report numbers of individuals at each stage of study—eg numbers potentially eligible, examined for eligibility, confirmed eligible, included in the study, completing follow-up, and analysed | ✔ |
|  |  | (b) Give reasons for non-participation at each stage | ✔ |
|  |  | (c) Use a flow diagram and include the figure number (preferably figure 1) or page number | ✔ |
| Descriptive data | 14* | (a) Give characteristics of study participants (eg demographic, clinical, social) and information on exposures and potential confounders | ✔ |
|  |  | (b) Indicate number of participants with missing data for each variable of interest | ✔ |
|  |  | (c) *Cohort study*—Summarise follow-up time (eg, average and total amount) | ✔ |
| Outcome data | 15* | *Cohort study*—Report numbers of outcome events or summary measures over time | ✔ |
|  |  | *Case-control study—*Report numbers in each exposure category, or summary measures of exposure | N/A |
|  |  | *Cross-sectional study—*Report numbers of outcome events or summary measures | N/A |
| Main results | 16 | (*a*) Give unadjusted estimates and, if applicable, confounder-adjusted estimates and their precision (eg, 95% confidence interval). Make clear which confounders were adjusted for and why they were included | ✔ |
|  |  | (*b*) Report category boundaries when continuous variables were categorized | ✔ |
|  |  | (*c*) If relevant, consider translating estimates of relative risk into absolute risk for a meaningful time period | ✔ |
| Other analyses | 17 | Report other analyses done—eg analyses of subgroups and interactions, and sensitivity analyses | ✔ |
| Discussion | | |  |
| Key results | 18 | Summarise key results with reference to study objectives | ✔ |
| Limitations | 19 | Discuss limitations of the study, taking into account sources of potential bias or imprecision. Discuss both direction and magnitude of any potential bias | ✔ |
| Interpretation | 20 | Give a cautious overall interpretation of results considering objectives, limitations, multiplicity of analyses, results from similar studies, and other relevant evidence | ✔ |
| Generalisability | 21 | Discuss the generalisability (external validity) of the study results | ✔ |
| Other information | | |  |
| Funding | 22 | Give the source of funding and the role of the funders for the present study and, if applicable, for the original study on which the present article is based | ✔ |

*Give information separately for cases and controls in case-control studies and, if applicable, for exposed and unexposed groups in cohort and cross-sectional studies.

**Note:** An Explanation and Elaboration article discusses each checklist item and gives methodological background and published examples of transparent reporting. The STROBE checklist is best used in conjunction with this article (freely available on the Web sites of PLoS Medicine at http://www.plosmedicine.org/, Annals of Internal Medicine at http://www.annals.org/, and Epidemiology at http://www.epidem.com/). Information on the STROBE Initiative is available at [www.strobe-statement.org](http://www.strobe-statement.org).
